# Chromosome-level genome assemblies of *Cutaneotrichosporon* spp. (Trichosporonales, Basidiomycota) reveal imbalanced evolution between nucleotide sequences and chromosome synteny

**DOI:** 10.1186/s12864-023-09718-2

**Published:** 2023-10-11

**Authors:** Yuuki Kobayashi, Ayane Kayamori, Keita Aoki, Yuh Shiwa, Minenosuke Matsutani, Nobuyuki Fujita, Takashi Sugita, Wataru Iwasaki, Naoto Tanaka, Masako Takashima

**Affiliations:** 1 Laboratory of Yeast Systematics, Tokyo NODAI Research Institute (TNRI), Tokyo University of Agriculture, 1-1-1 Sakuragaoka, Setagaya, Tokyo, 156-8502 Japan; 2 Department of Molecular Microbiology, Faculty of Life Sciences, Tokyo University of Agriculture, 1-1-1 Sakuragaoka, Setagaya, Tokyo, 156-8502 Japan; 3 NODAI Genome Research Center, Tokyo University of Agriculture, 1-1-1 Sakuragaoka, Setagaya, Tokyo 156-8502 Japan; 4 Department of Microbiology, Meiji Pharmaceutical University, 2-522-1 Noshio, Kiyose, Tokyo, 204-8588 Japan; 5 Department of Integrated Biosciences, Graduate School of Frontier Sciences, The University of Tokyo, Kashiwa, Chiba 277-0882 Japan

**Keywords:** Comparative genomics, Genome evolution, Chromosome rearrangement, Taxonomy, *Cutaneotrichosporon*, Yeast, Basidiomycota

## Abstract

**Background:**

Since DNA information was first used in taxonomy, barcode sequences such as the internal transcribed spacer (ITS) region have greatly aided fungal identification; however, a barcode sequence alone is often insufficient. Thus, multi-gene- or whole-genome-based methods were developed. We previously isolated Basidiomycota yeasts classified in the Trichosporonales. Some strains were described as *Cutaneotrichosporon cavernicola* and *C. spelunceum*, whereas strain HIS471 remained unidentified. We analysed the genomes of these strains to elucidate their taxonomic relationship and genetic diversity.

**Results:**

The long-read-based assembly resulted in chromosome-level draft genomes consisting of seven chromosomes and one mitochondrial genome. The genome of strain HIS471 has more than ten chromosome inversions or translocations compared to the type strain of *C. cavernicola* despite sharing identical ITS barcode sequences and displaying an average nucleotide identity (ANI) above 93%. Also, the chromosome synteny between *C. cavernicola* and the related species, *C. spelunceum*, showed significant rearrangements, whereas the ITS sequence identity exceeds 98.6% and the ANI is approximately 82%. Our results indicate that the relative evolutionary rates of barcode sequences, whole-genome nucleotide sequences, and chromosome synteny in *Cutaneotrichosporon* significantly differ from those in the model yeast *Saccharomyces*.

**Conclusions:**

Our results revealed that the relative evolutionary rates of nucleotide sequences and chromosome synteny are different among fungal clades, likely because different clades have diverse mutation/repair rates and distinct selection pressures on their genomic sequences and syntenic structures. Because diverse syntenic structures can be a barrier to meiotic recombination and may lead to speciation, the non-linear relationships between nucleotide and synteny diversification indicate that sequence-level distances at the barcode or whole-genome level are not sufficient for delineating species boundaries.

**Supplementary Information:**

The online version contains supplementary material available at 10.1186/s12864-023-09718-2.

## Background

Fungal microorganisms are among the most ubiquitous life forms on Earth. Since fungi have fewer morphological traits than metazoans or land plants, molecular identification is widely used in fungal taxonomy. In fungal systematics, the internal transcribed spacer (ITS) sequence is the preferred barcode used for conventional molecular identification [1, 2]; however, the ITS sequence alone is not always sufficient for recognizing species [3]. Therefore, the D1/D2 domain of the LSU rRNA gene is employed in combination with the ITS region to more accurately identify yeast species [4, 5].

The whole genome-based method is a more comprehensive way to identify species using molecular technology. DNA-DNA hybridization (DDH) is a traditional whole genome-based approach for species delineation of microorganisms, including fungi and bacteria [6, 7]. In the case of bacteria, the average nucleotide identity (ANI) method was developed to mimic DDH using genome sequence information [8]. Another method, the genome blast distance phylogeny (GBDP) method, is also used in combination with the ANI method [9]. Using whole genome data for fungal identification is not yet widespread but can be a powerful tool [10].

Yeasts are unicellular fungi that evolved convergently from filamentous fungi in the Ascomycota and Basidiomycota [11]. Currently, approximately 2000 yeast species are described [12], yet, there still are many yeasts whose taxonomic positions are not fully known.

Tremellomycetes is a class of Basidiomycota that includes many yeast species, such as *Cryptococcus* spp. and *Trichosporon* spp. [13]. We previously reported the identity of several strains of Tremellomycetes yeasts found in bat-inhabited caves in Japan as *Trichosporon* [14]. Several strains were later described as *Cutaneotrichosporon cavernicola*, with strain HIS019 designated as the type strain [15]. Strains HIS002, HIS631, and HIS641 were also described as *C. cavernicola* with HIS019 (hereafter identified as “*C. cavernicola* standard strains”). HIS016 was designated as type material during the valid description of *Trichosporon spelunceum* [16] (previously HIS016 was associated with the invalidly described name *Trichosporon shinodae*). Later *T. spelunceum* was recombined as *Cutaneotrichosporon spelunceum* [17].

Here, we report the identity of strain HIS471, which has not been previously identified. We sequenced the complete genomes of these strains to elucidate their genomic backgrounds. We compared barcode sequences, whole genome similarity, and chromosome synteny of these strains and assessed their similarity and differences compared to the well-studied model yeast genus *Saccharomyces*.

## Results

### Morphology and barcode sequence of strain HIS471

Both yeast and hyphal cells were observed during vegetative growth of strain HIS471(Fig. S1A, B), as was the case for the *C. cavernicola* standard strains [15]. The identical ITS sequence also supported a close relationship with the *C. cavernicola* standard strains (Fig. S1C); however, the D1/D2 region had four base substitutions (Fig. S1D) that may affect the secondary structure of the transcribed RNA (Fig. S1E, F), suggesting strain HIS471 is a different species [5]. In Trichosporonales, a similar example was reported for *Apiotrichum domesticum* and *A. montevideense* (formerly *Trichosporon domesticum* and *T. montevideense*, respectively); the strains have identical sequences in the ITS region but two nucleotide differences in the D1/D2 domain, and are recognised as separate species based on DNA-DNA relatedness analysis [18].

### Sequencing and assembly results

We sequenced and assembled the genomes of four *C. cavernicola* standard strains (HIS002, HIS019, HIS631, and HIS641), one strain whose phenotype resembles *C. cavernicola* (HIS471), and the type strain of *C. spelunceum* (HIS016). The assembly size of each strain was approximately 20 Mb, similar to the estimated genome size calculated from Illumina genomic reads (Table 1). The genome size of *C. spelunceum* HIS016 is slightly smaller than the *C. cavernicola* standard strains and strain HIS471. The number of contigs presumed to correspond to nuclear genomes was seven to twelve. The predicted number of repetitive sequences was as little as approximately 2% of the total assembly, a lower level than that of model yeast species *S. cerevisiae* and the well-studied Tremellomycetes yeast *Cr. neoformans* (Table 1, S1). The BUSCO assessment found approximately 90 to 94% of conserved single-copy genes with 0.3 to 0.4% duplication in the draft genomes. These values indicate a high assembly completeness of haploid genomes (Table 1).

**Table 1:** Summary of genome assemblies

Species			*C. cavernicola*				*C.* aff. *cavernicola*	*C. spelunceum*
Strain			HIS002	HIS019	HIS631	HIS641	HIS471	HIS016
Estimated genome size (Mbp)			20.31	19.60	19.82	19.36	20.06	18.79
Nuclear genome								
	Assembly size (Mbp)		20.20	20.24	20.23	20.23	20.60	19.16
	No. of contigs		7	8	7	8	8	12
	GC %		58.49	58.48	58.49	58.49	57.79	59.71
	Repeat content (%)		2.08	2.10	2.05	2.08	2.20	2.04
	BUSCO completeness (%)							
		Complete single-copy	89.7	92.5	92.1	92.1	92.2	93.7
		Complete and duplicated	0.3	0.4	0.4	0.4	0.4	0.3
		Fragmented	1.6	0.9	0.9	1.1	0.8	1.6
		Missing	8.4	6.2	6.6	6.4	6.6	4.4
Mitochondrial genome size (bp)			40,463	40,463	40,460	40,460	42,028	42,135

**Fig. 1:**
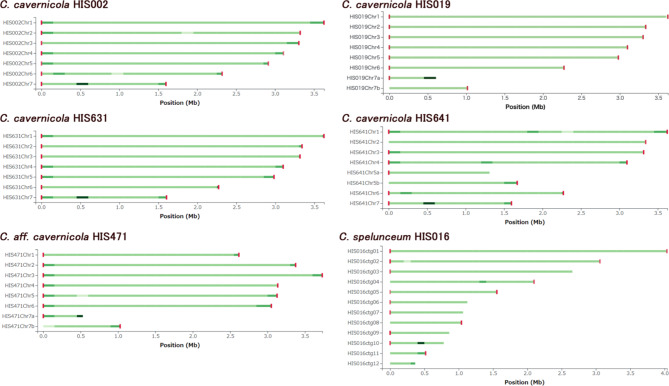
Chromosome continuity of genomes assemblies. Telomere sequence and sequencing depth was illustrated using Tapestry 1.0.0. Red rectangles at the termini stand for telomere repeat sequences (CCCCTAA/TTAGGGG). The intensity of the green lines indicates the depth of sequencing reads. The dark coloured region on Chr.7 in the genomes of HIS002, HIS019, HIS631, HIS641, and HIS471, and on ctg.10 in the genome of HIS016 correspond to rDNA repeats

All seven contigs of *C. cavernicola* HIS002 and HIS631 have telomere sequences on both ends (Fig. 1). Although the number of ribosomal DNA (rDNA) repeats may not be precise, these contigs are likely to be equivalent to chromosomes. The chromosome number of species in Trichosporonales has not yet been experimentally determined but our result suggests that the chromosome number of *C. cavernicola* is seven. Hence, we named the contigs as chromosomes 1 to 7 based on their length. The contigs of other strains may not have full continuity as whole chromosomes but are nearly appropriate for chromosome assembly. Thus, we sorted and named these contigs as corresponding to specific chromosomes except for HIS016, whose contigs could not be assigned to chromosomes.

The self-synteny plot of the *Cutaneotrichosporon* genome showed no obvious centromeric repeats, whereas the reference *Cryptococcus* genome showed repetitive palindromic sequences corresponding to centromeres in each chromosome (Fig. S2). Thus, *Cutaneotrichosporon* may be holocentric or have very short centromeres like *Saccharomyces* [19].

Gene model annotation based on both *de novo* prediction and RNA-seq hints predicted approximately 7,300 to 7,800 genes (Table 2), a relatively smaller number than in *C. oleaginosus* [20] and *Trichosporon asahii* [21], but in the range for Trichosporonales spp. [15]. The number of genes in *C. spelunceum* HIS016 was smaller than in other strains, as was the genome size. The completeness of BUSCO single-copy genes was raised to approximately 95 to 97%.

**Table 2:** Summary of predicted genes

Species		*C. cavernicola*				*C.* aff. *cavernicola*	*C. spelunceum*
Strain		HIS002	HIS019	HIS631	HIS641	HIS471	HIS016
Number of genes		7,796	7,754	7,758	7,738	7,698	7,309
Number of transcripts		7,908	7,866	7,871	7,842	7,820	7,411
BUSCO completeness (%)							
	Complete single-copy	94.5	96.6	96.3	95.5	94.2	95.6
	Complete and duplicated	0.4	0.4	0.4	0.4	0.4	0.4
	Fragmented	0.8	0.3	0.5	1.1	1.1	0.8
	Missing	4.3	2.7	2.8	3.0	4.3	3.2

### Comparison of nuclear genomes

**Fig. 2:**
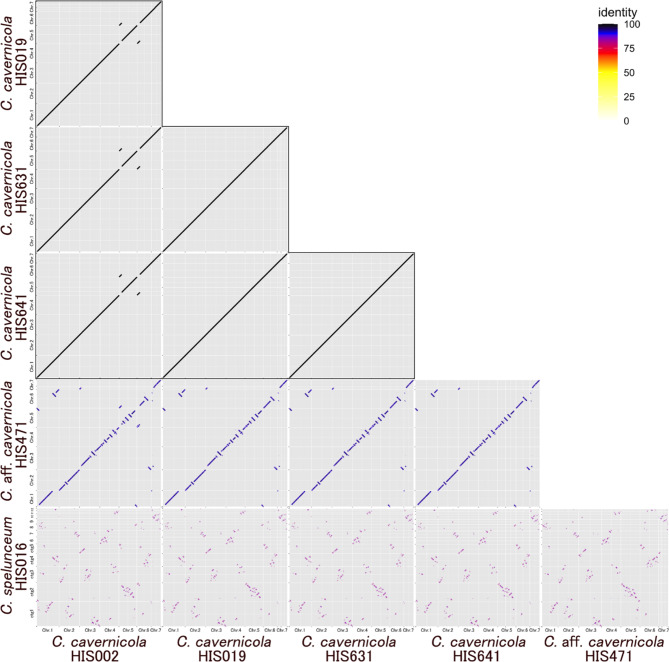
Plots of chromosome synteny based on pairwise BLASTN alignment among *Cutaneotrichosporon* strains. The line colour reflects the percentage of nucleotide identity in the alignment as shown in the legend

We compared the genome synteny of sequenced strains. The four genomes of *C. cavernicola* standard strains showed a consistent pattern of genome synteny except for HIS002, which has one translocation between chromosome 5 and chromosome 6 compared to other *C. cavernicola* strains (Fig. 2, upper three rows, Fig. S3). In contrast, the genome of strain HIS471 showed many chromosome rearrangements compared to *C. cavernicola* standard strains, suggesting more than ten translocations or inversions (Fig. 2, fourth row from the top, Fig. S3). Moreover, the genome of *C. spelunceum* HIS016 showed highly fragmented synteny that made the identification of corresponding chromosomes difficult (Fig. 2, bottom row, Fig. S3). The number of chromosome rearrangements between *C. cavernicola* standard strains and strain HIS471 seems higher than interspecific differences in the model yeast genus *Saccharomyces* (Fig. 2, fourth row, Fig. S4). Also, the number of chromosome rearrangements between *C. cavernicola* and *C. spelunceum* is much higher than intragenic differences in *Saccharomyces* (Fig. 2, bottom row, Fig. S4).

In contrast to highly diverged chromosome synteny, sequences of the standard fungal genetic barcode, ITS, are highly conserved within all *Cutaneotrichosporon* species (Fig. S5). Specifically, the strain HIS471 harbours an ITS sequence identical to that of *C. cavernicola* standard strains (Fig. S5). According to the current guidelines the same ITS sequence belong to the same species [5]. However, here the conserved barcode sequences are not consistent with the differences observed in chromosome synteny.

Comparison of secondary barcoded genes showed that *C. cavernicola* HIS019, *C*. aff. *cavernicola* HIS471, and *C. spelunceum* HIS016 had different exon-intron structures in *ACT1*, *TEF1*, and *RPB2* (Table S2). Therefore, the genomic sequences of coding genes differ to some extent among these strains. Considering that splicing variations can significantly impact alignment, they should be taken into account in barcoding.

### Quantification of differences in genomes using different criteria

**Fig. 3:**
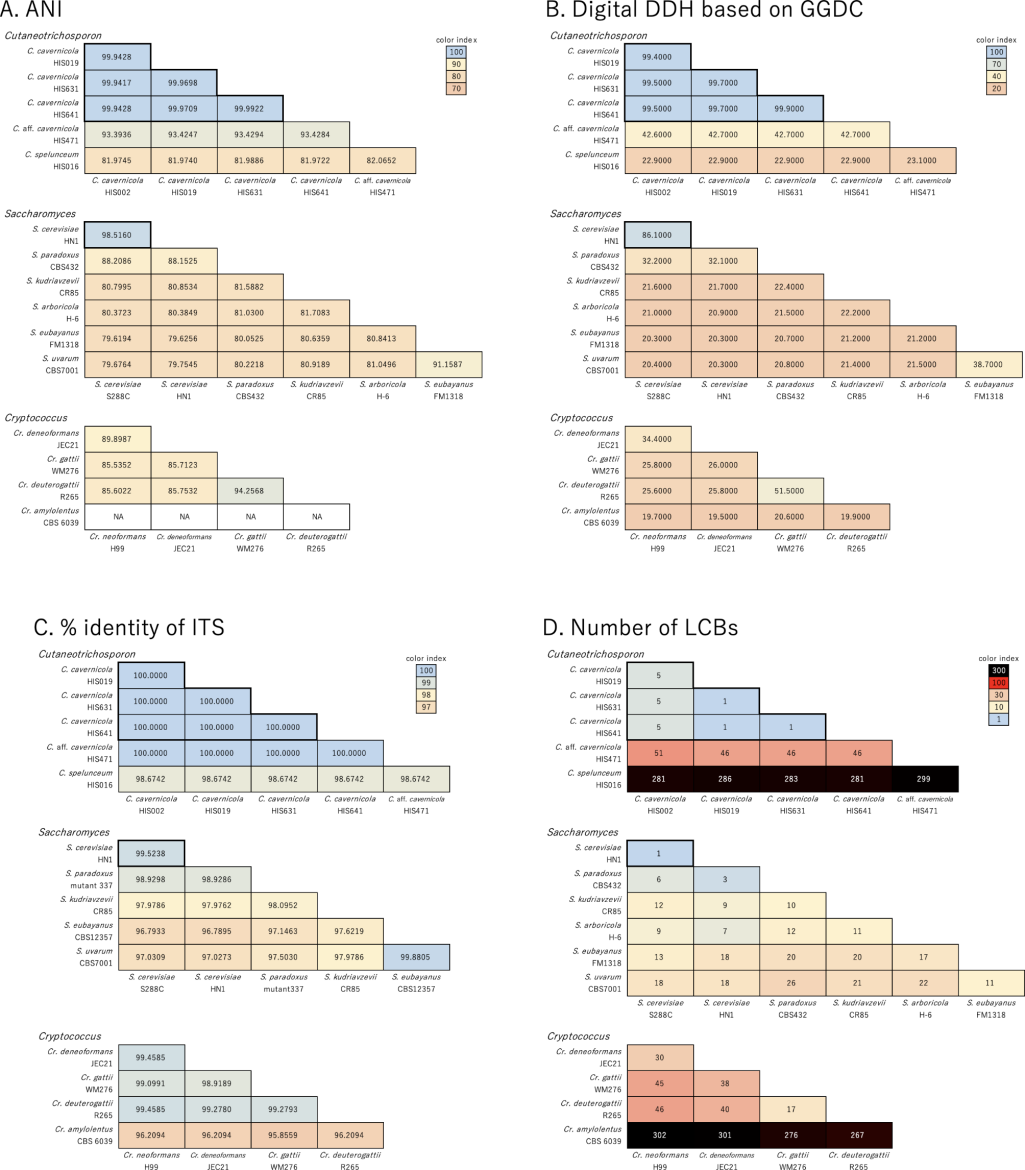
Genome similarities based on multiple criteria among *Cutaneotrichosporon* strains compared with reference *Saccharomyces* and *Cryptococcus*. Thick-bordered areas in the *Cutaneotrichosporon* and *Saccharomyces* panels indicate intraspecific comparisons among the *C. cavernicola* standard strains and *S. cerevisiae*, respectively. Box colours identify identical genomes (blue) and the most distant interspecific comparison (orange) in *Saccharomyces*. **A**; ANI score. **B**; GBDP score calculated with GGDC. The scores for formula 2 are shown according to the recommendation in Henz et al. [9], and scores by all three formulae are shown in Fig. S6. **C**; Percentage identity in the ITS sequence. **D**; Number of LCBs with a minimum weight of 10 kb

To assess the genetic diversity of *Cutaneotrichosporon* from multiple perspectives, we quantified similarities in the whole genome sequences, barcode sequences, and chromosome synteny and compared their values with those of *Saccharomyces* and *Cryptococcus*. To estimate the similarity of whole-genome nucleotide sequences, we calculated the ANI and GBDP scores. Among *C. cavernicola* standard strains, both ANI and GBDP scores were more than 99.9%, indicating that the whole-genome nucleotide sequences of these strains are quite similar (Fig. 3A, B, S6). In comparing the *C. cavernicola* standard strains and *C. spelunceum*, ANI values were approximately 82.0%, and GBDP scores were lower than 30%, showing a certain distance among the genomic sequences. In the comparison between *C. cavernicola* standard strains and the strain HIS471, ANI values were approximately 93.4%, and GBDP scores were approximately 42.7% by formula 2 and approximately 85–95% by other formulae (Fig. 3B, S6). When we compared these scores with that of *Saccharomyces*, differences in the whole genome sequences among *C. cavernicola* standard strains were within the range of intraspecific diversity in *S. cerevisiae*. The whole-genome similarity between the *C. cavernicola* standard strains and *C. spelunceum* was comparable to the interspecific similarity in *Saccharomyces*. The similarity between the *C. cavernicola* standard strains and strain HIS471 was intermediate between the intraspecific and interspecific similarities of *Saccharomyces*, although the GBDP scores differed primarily by formulae in both *Cutaneotrichosporon* and *Saccharomyces*.

The ITS sequences of the *C. cavernicola* standard strains and strain HIS471 were identical, and the identity between these strains and *C. spelunceum* was approximately 98.7% (Fig. 3C). Compared to *Saccharomyces*, the difference in ITS sequences between the *C. cavernicola* standard strains and HIS471 was at the intraspecific diversity level in *S. cerevisiae*. In contrast, the difference between *C. cavernicola* and *C. spelunceum* was like that of *S. cerevisiae* and its closest species, *S. paradoxus*. Hence, the ITS-based genetic distance among *Cutaneotrichosporon* strains was estimated to be smaller than that obtained by the whole-genome-based prediction. When compared to *Cryptococcus*, the differences between *Cr. amylolentus* and *Cr. gattii/neoformans* species complex (*Cr. neoformans*, *Cr. deneoformans*, *Cr. gattii*, and *Cr. deuterogattii*) are so pronounced that it is not possible to calculate ANI. In addition, the similarities calculated using GGDC or ITS were also lower than those observed in *Cutaneotrichosporon* or *Saccharomyces*. This suggests that the genus *Cryptococcus* includes more diverse species. However, the degree of difference within *Cr. gattii/neoformans* species complex was similar to intrageneric differences observed in *Cutaneotrichosporon* or *Saccharomyces*. In the *Cr. gattii/neoformans* species complex, the ratio of ITS sequence conservation to whole-genome sequence conservation more closely resembled that of *Cutaneotrichosporon* than *Saccharomyces* (Fig. 3).

We also quantified chromosome rearrangements. We chose the number of locally colinear blocks (LCBs) as an index of chromosome rearrangement. If no threshold was set for a minimum LCB weight, non-specific LCBs caused by repetitive sequences, especially telomeric repeats, were counted (Fig. S7). Therefore, we calculated the number of LCBs with the minimum LCB weight set at 10 kb to assess gross chromosome rearrangements. In this comparison, the number of LCBs between *C. cavernicola* standard strains and strain HIS471 was approximately 50, and the number of LCBs between *C. cavernicola* and *C. spelunceum* was approximately 280 (Fig. 3D). In contrast, all LCB values among *Saccharomyces* species were less than 30. Hence, chromosome synteny between the *C. cavernicola* standard strains and strain HIS471 was less conserved than the interspecific conservation in *Saccharomyces*, and the synteny between *C. cavernicola* and *C. spelunceum* was much less conserved. Also, the number of LCBs between *C. cavernicola* and HIS471 was greater than between *Cr. gattii* and *Cr. deuterogattii* while the sequence similarity was comparable. A similar trend was observed in the comparison between *C. cavernicola* - *C. spelunceum* and *Cr. neoformans* - *Cr. gattii*.

These results suggest that the evolution rate of barcode sequences, whole genome sequences, and chromosome synteny is different between *Cutaneotrichosporon*, *Saccharomyces*, and *Cryptococcus*.

### Genes and synteny of mitochondrial genomes

**Fig. 4:**
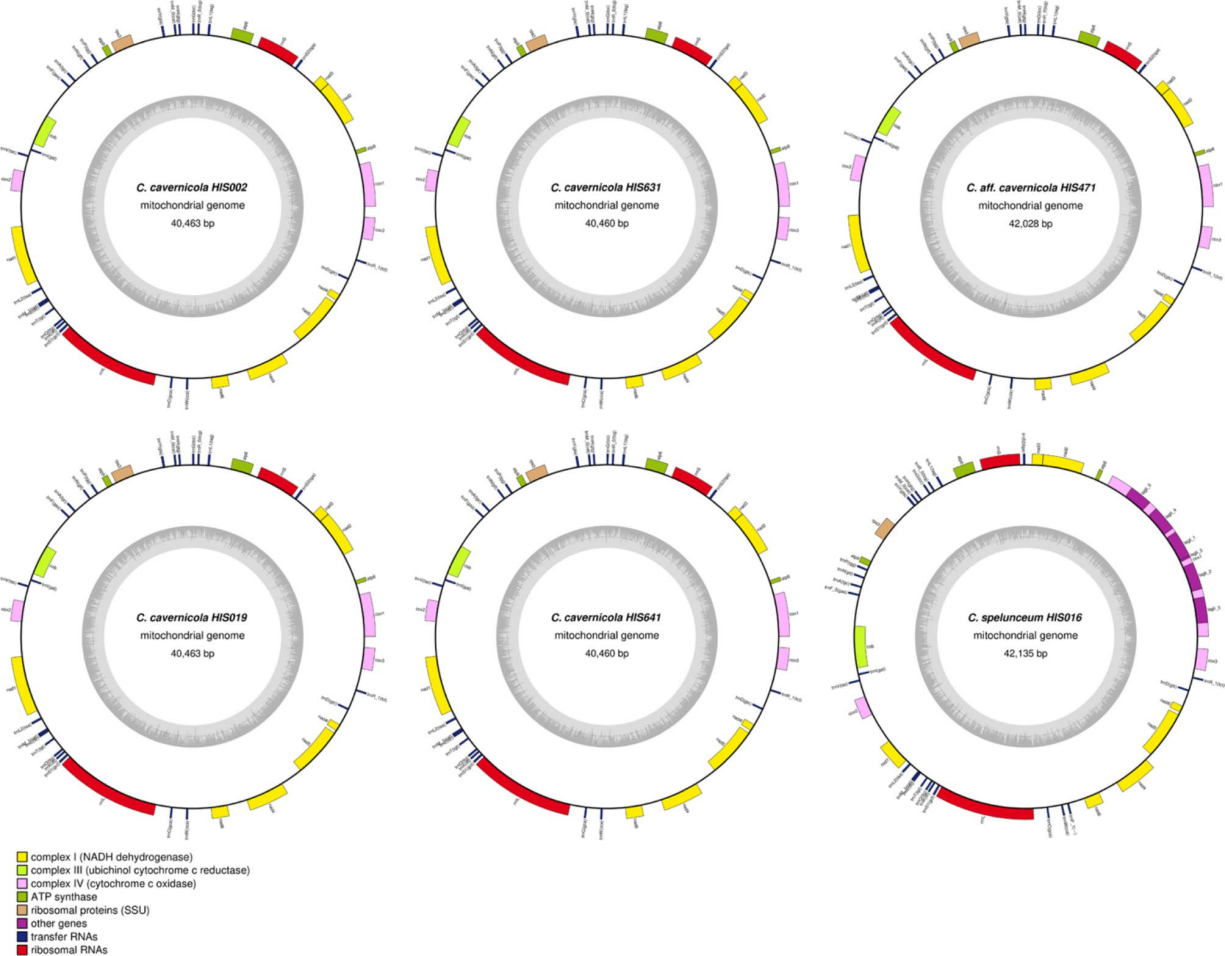
Mitochondrial genomes of *Cutaneotrichosporon* strains. Genes projecting outward from the outer circles indicate genes transcribed in the forward direction; genes projecting inward from the outer circles indicate genes transcribed in the reverse direction. Gene families are identified by colour as shown in the legend. The inner circles represent the GC content of the sequences

**Fig. 5:**
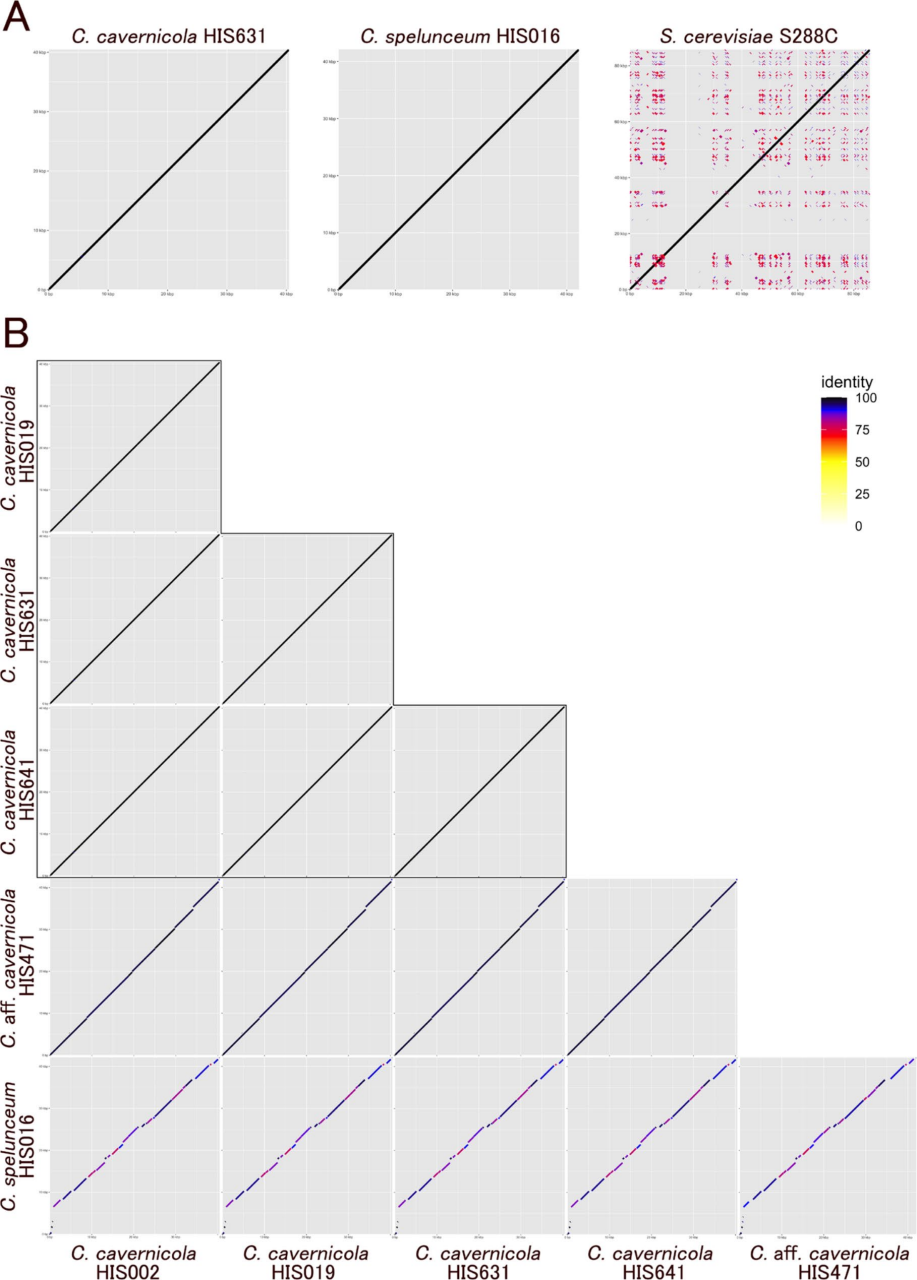
Plots of mitochondrial genome synteny based on pairwise BLASTN alignments. Line colour reflects the percentage of nucleotide identity in the alignment as shown in the legend. **A**; Self synteny of *C. cavernicola* HIS631, *C. spelunceum* HIS016, and the reference *S. cerevisiae* S288C. **B**; Pairwise synteny plots among *Cutaneotrichosporon* mitogenomes

Next, we checked the mitochondrial genomes of *Cutaneotrichosporon* strains. We obtained single circular mitochondrial DNA sequences with sizes ranging from approximately 40 to 42 kb (Table 1). All 15 mitochondrial core genes (*COX1*, *COX2*, *COX3*, *COB*, *ATP6*, *ATP8*, *ATP9*, *NAD1*, *NAD2*, *NAD3*, *NAD4*, *NAD4L*, *NAD5*, *NAD6*, *RPS3*) were found (Fig. 4). Only *C. spelunceum* had five introns encoding a putative LAGLIDADG homing endonuclease in the *cox1* gene (Fig. 4). Homologous sequences of those introns were not found in the mitochondrial or nuclear genomes of other strains. Mitochondrial genomes of *Cutaneotrichosporon* lacked repetitive sequences found in *Saccharomyces*, as well as for the nuclear genomes (Fig. 5A). However, unlike nuclear genomes, only indels, including the previously mentioned introns, were found in the comparison of mitochondrial genomes and no inversions or translocations were found (Fig. 5B).

## Discussion

In this study, we sequenced the whole genome of six strains of *Cutaneotrichosporon* Basidiomycota yeast. Our chromosome-level assembly revealed that *C. cavernicola*, *C. spelunceum* and strain HIS471 have experienced many chromosome rearrangements, whereas the ITS sequences remain highly conserved with ANI scores greater than 80% (Fig. 3, S6). Comparative analyses showed that the balance between differentiation in nucleotide sequence and chromosome synteny in *Cutaneotrichosporon* was mainly different from that of the model yeast, *Saccharomyces* (Fig. 3). In addition, the degree of chromosome inversions or translocations between HIS471 and the *C. cavernicola* standard strains occurred more frequently than in *Candida albicans* and *Ca. dubliniensis* as reported by Li et al. [22]. These results suggest that the rate of nucleotide sequence evolution and levels of chromosome synteny may differ among fungal clades. It is not clear, however, whether nucleotide mutations are repressed or chromosome rearrangements are accelerated in *Cutaneotrichosporon* compared to *Saccharomyces*. It is also paradoxical that *Cutaneotrichosporon* genomes harbour very few repetitive elements (Table S1) that can potentially cause chromosome rearrangements [23, 24]. Several mechanisms to prevent repeat-induced chromosome rearrangement have been reported [25]. *Cutaneotrichosporon* could have lost or reduced such processes due to the loss of repeats. The real entity causing the difference is unclear, and further research is required.

From the viewpoint of taxonomy, identifying the criteria applicable to species delineation is worthy of investigation. Barcode sequences are the most popular form for conventional molecular identification, especially for multicellular organisms [26, 27]. For fungal classification, the ITS sequence in the rDNA region is the most frequently used DNA barcode [1]; however, the range of ITS variation within a single species differs depending on the taxon [28]. Our results suggest that asynchronous differentiation between the ITS sequences and whole-genome sequences or chromosome conformation may be the reason for intraspecific ITS diversity. As for the GBDP method, genome-to-genome distance scores showed significant differences depending on the formula used in both *Cutaneotrichosporon* and *Saccharomyces*, although the genome assemblies are almost complete. This result contrasts with a case in bacteria in which comparison of well-assembled genomes resulted in similar scores by all formulae [9]. The reason for obtaining different scores with different formulae is not clear. It could be due to differences in genome characteristics between prokaryotes and eukaryotes, such as different gene density, different GC content or the existence of introns. Further studies are needed to elucidate the reason for the differences and to optimize this method for use in eukaryotic genome comparisons.

In the case of prokaryotes, barcode sequence similarity does not always correlate with whole-genome similarity [29]. Our results showed that this discrepancy is also true in eukaryotes. Moreover, our results also showed that chromosome synteny does not always correlate with either barcode sequence or whole-genome similarity. Genome rearrangement is known to cause mating infertility and speciation in Ascomycota, such as *Saccharomyces* and *Schizosaccharomyces* [30–32]. If this mechanism is universal, chromosome synteny should be considered the determinant of biological species, i.e., the boundary of genetic pools separated by reproductive isolation. If so, neither barcode sequences nor whole-genome similarity might be sufficient for defining a species.

Discoveries in this study were achieved by chromosome-level genome assembly. Our results also revealed the impact of complete genome sequencing as a powerful tool for taxonomy studies, equal to investigating biological traits. The continued accumulation of high-quality genomic data will contribute toward elucidating how evolution and the ecology of fungal species are related.

## Conclusions

Our chromosome-level assembly of *Cutaneotrichosporon* genomes and comparative study with *Saccharomyces* revealed that the ratio of conservativeness among barcode sequences, whole genome sequences, and chromosome synteny are different among fungal groups. Hence, the rate of nucleotide sequence evolution and chromosome synteny may not be uniform among species, but lineage-specific mutation repression or acceleration may exist.

Currently, genomic information is becoming more important for taxonomy; however, our results revealed that estimated genetic distances could differ substantially based on which criteria are used: barcode sequences, whole-genome sequences, or chromosome synteny. Our study suggests that a comprehensive assessment, not based on a single criterion, may be the best approach to use for genome-based taxonomy.

## Methods

### Fungal materials

The yeast strains were isolated as described by Sugita et al. 2005 and Takashima et al. 2020, and maintained at Meiji Pharmaceutical University. The strain HIS019 is also available at the Riken Bioresource Center as JCM 12,590. For genomic and RNA sequencing, cells were incubated for one to three days in a YM liquid medium (10 g glucose, 3 g yeast extract, 3 g malt extract, and 5 g peptone per litre) at 25 °C, with shaking at 100 rpm.

### Isolation of genomic DNA and RNA

For genomic DNA extraction, cultured cells were lysed with Westase (Ozeki, Japan) following the manufacturer’s instructions for *Saccharomyces cerevisiae*. Genomic DNA was extracted following Westase’s protocol provided by the distributor (Takara, Japan). Isolated DNA was purified using Genomic-tip 20/G columns (Qiagen, Netherlands). For RNA extraction, cells were twice disrupted using a vortex mixer with glass beads for 30 s. RNA was extracted using a NucleoSpin RNA Plant and Fungi Mini Kit (Macherey-Nagel, Germany) following the manufacturer’s instructions.

### NGS library construction and sequencing

Genomic DNA was sequenced with Nanopore sequencers (Oxford Nanopore Technologies, UK). The genome structure of HIS019 and HIS471 was confirmed with PacBio sequencing (Pacific Biosciences, USA). For long-read sequencing, genomic DNA (6 mg) was treated with a Short-Read Eliminator Kit XS (Circulomics) to remove fragments < 10 kbp, and libraries were prepared using a Rapid Barcoding Sequencing Kit (SQK-RBK004, Oxford Nanopore Technologies). Sequencing was performed on the MinION (Sample HIS002 and HIS019) and GridION X5 (Sample HIS631, HIS641, HIS016, HIS471) systems using eight R9.4 flow cells. PacBio library construction and sequencing with Sequel II (Pacific Biosciences, USA) was outsourced (Takara Bio, Japan). Illumina paired-end genomic libraries with insert sizes of 300–350 bp were constructed with a Nextera DNA Flex Library Prep Kit (Illumina, USA). The libraries were sequenced with the NextSeq 500/550 Mid Output Kit v2.5 (Illumina, USA) for 151 bp from both ends. Illumina RNA-seq libraries were constructed with the NEBNext Ultra II Directional RNA Library Prep Kit (New England Biolabs, USA) for Illumina and sequenced with the NextSeq 500/550 Mid Output Kit v2.5 (Illumina, USA) for 151 bp from both ends.

### Genome assembly, assessment, and annotation

The genome sizes of strains were estimated with GenomeScope2.0 [33] following k-mer (k = 21) counting with Jellyfish 2.3.0 [34] using Illumina genomic reads. Nanopore genomic reads were assembled with Canu 2.2 [35]. Draft genome assemblies from Nanopore reads were polished with Pilon 1.22 [36] after mapping the Illumina genomic reads with Bowtie2 2.4.5 [37]. PacBio Hifi reads were assembled with Hifiasm 0.16.1-r375 [38]. Contigs, other than mitochondrial contigs or short fragments of rDNA repeats, were regarded as nuclear genome contigs. The order and direction of contigs were manually sorted with SeqKit 2.2.0 [39]. Assembly completeness was assessed with BUSCO 5.4.2 [40] with fungi_odb10 (n = 758) selected as the reference database. Telomeres were searched with Tapestry 1.0.0 [41] with the sequence TTAGGGG functioning as the telomere repeat sequence.

Repetitive sequences were predicted and soft-masked with RepeatMasker 4.1.1 [42] using a custom repeat model constructed with RepeatModeler 2.0.1 [43]. Illumina RNA-seq reads were mapped to draft genomes with HiSat2 2.2.1 [44]. Genes were predicted with BRAKER 2.1.6 [45] using the combination of GeneMark-ES 4.69 [46] and Augustus 3.4.0 [47]. Mapped RNA-seq reads were used as an Augustus hint file.

### Mitochondrial genome construction and annotation

Mitochondrial contigs were searched from the draft assembly with NCBI-blast 2.2.31+ [48] using the mitochondrial sequence of *S. cerevisiae* as a query. Identified mitochondrial contigs were manually adjusted so that contigs start from the start codon of COX1 as the forward strand. If a mitochondrial contig was not found in the draft assembly, the mitochondrial genome was assembled from reads mapped to the mitochondrial genome of other strains with Unicycler 0.5.0 [49]. Gene models were primarily annotated with two methods; MITOS2 [50] (accessed 2023.02.07) with the “RefSeq fungi” dataset as a reference and “mold mitochondrial genetic code” (genetic code 4) and AGORA [51] (accessed 2023.02.07) with the mitochondrial genome of *Tremella fuciformis* (NC_036422) as a reference. Annotations were manually edited using the above-predicted information. Images were drawn with OGDRAW [52].

### Comparative analyses

Syntenic regions of genomic sequences were searched using NCBI-BLAST 2.2.31+ [48] with one assembly as the query and the other as the database. Visualization was archived with our own scripts (see the “Availability of data and materials” section) prepared using R 4.2.2 [53]. Chromosome synteny was also confirmed with Mauve 2015-2-25 [54]. Sequences of ITS regions were extracted from whole-genome assemblies with SeqKit 2.2.0 [39] (seqkit amplicon command) using ITS1 and ITS4 primer sequences [55]. The positions of the ITS sequences used are shown in Table S3. ITS sequences were aligned with MAFFT 7.511 [56]. ANI was calculated with fastANI 1.33 [57]. The GBDP scores were calculated with the Genome-to-Genome Distance Calculator (GGDC) 3.0 [58, 59]. The values from Formula 2, one of three formulas (Formula 1, based on high-scoring segment pairs per total length; Formula 2, based on identity per high-scoring segment pairs; and Formula 3, based on identities per total length), are displayed in Fig. 3, with all equation values available in Fig. S6. The number of LCBs was counted with Mauve 2015-2-25 [54].

### Reference genomes

Assembly genomes of *S. cerevisiae* S288C (GCF_000146045.2) [60], *S. cerevisiae* HN1 (GCA_903819125.2) [61], *S. paradoxus* CBS 432^T^ (GCF_002079055.1) [62], *S. kudriavzevii* CR85 (GCA_003327635.1) [63], *S. arboricola* H-6T (GCF_000292725.1) [64], *S. eubayanus* FM1318 (GCF_001298625.1) [65], *S. uvarum* CBS 7001 (GCA_947243805.1), *Cr. neoformans* H99 (GCA_011801205.1) [66], *Cr. deneoformans* JEC21 (GCF_000091045.1 [67], registered as “*Cr. neoformans*” in GenBank but is currently classified as *Cr. deneoformans* [68]), *Cr. gattii* WM276 (GCF_000185945.1) [69], *Cr. deuterogattii* R265 (GCA_002954075.1) [69], and *Cr. amylolentus* CBS6039 (GCF_001720205.1) [70] were downloaded from the NCBI website [71]. Since the whole ITS sequence was absent from GCF_002079055.1, GCF_001298625.1, and GCF_001720205.1, assembly genomes of *S. paradoxus* mutant337 (CP081978.2) and *S. eubayanus* CBS 12,357^T^ (GCA_003327605.1) [72] were used to extract the ITS sequences. Additionally, we used the ITS sequence of *Cr. amylolentus* CBS 6039 as deposited in GenBank (NR_111372.1) [73]. To calculate the LCB, the reference assemblies of *Cr. gattii/neoformans* species complex were sorted to align with the RefSeq data of *Cr. deneoformans* JEC21 (GCF_000091045.1) [67], as shown in Table S4, because the chromosomes were not aligned in the corresponding order or orientation.

### Electronic supplementary material

Below is the link to the electronic supplementary material.

**Fig. S1**. Characteristics of Cutaneotrichosporon sp. HIS471. A, B; Microscopic images of strain HIS471. Cells were incubated on corn meal agar medium (Nissui, Japan) at 20 °C for four days, and observed using a BX53 compound microscope with an UPlansXApo 40? objective lens (Olympus, Japan). Bars; 20 m. C, D; Pairwise alignment of strain HIS019 (C. cavernicola type) and strain HIS471. C and D represent the ITS region and the D1/D2 region, respectively. The polymorphic sites are indicated by arrows. E, F; The possible RNA secondary structure of D1/D2 region of HIS019 (E) and HIS471 (F) predicted with the minimum free energy (MFE) method. **Fig. S2**. Self-synteny plots of C. cavernicola and Cryptococcus neoformans genomes. Self-synteny plots of C. cavernicola HIS019 and reference Cryptococcus neoformans H99 (GCA_011801205.1) genomes. The plot of the C. cavernicola genome shows no visible repeats, in contrast to the plot of the Cr. neoformans genome, which shows repetitive palindromes (which appear as “X” in the figure) corresponding to the centromeres in each chromosome. **Fig. S3**. Mauve alignment of Cutaneotrichosporon genomes. Chromosome synteny of Cutaneotrichosporon visualized with Mauve 2015-2-25. Each coloured block represents locally colinear blocks (LCBs). **Fig. S4**. Chromosome synteny of Saccharomyces. BLASTN-based chromosome synteny of the reference model yeast Saccharomyces. Line colour reflects the percentage of nucleotide identity in the alignment as shown in the legend. **Fig. S5**. Alignment of ITS sequences of Cutaneotrichosporon strains. Multiple alignment of ITS sequences of Cutaneotrichosporon strains. The ITS sequences were extracted from assembly genomes with the SeqKit amplicon. The polymorphic sites are indicated by arrows. **Fig. S6**. GBDP scores calculated by all three formulae of GGDC. The GBDP scores among Cutaneotrichosporon and among reference Saccharomyces and Cryptococcus calculated by using three formulae with the genome-to-genome distance calculator (GGDC). Blue boxes represent identical genomes and orange boxes represent the most distant interspecific comparison in the reference genomes. **Fig. S7**. Satellite syntenies in reference Saccharomyces caused by repetitive sequences. Chromosome synteny between reference S. cerevisiae and S. paradoxus. A; BLAST-based synteny visualization. Red arrowheads represent satellite syntenies caused by telomeric repeats. B; Synteny visualized with Mauve 2015-2-25 alignment. Red arrowheads represent LCBs from satellite syntenies of repetitive sequences.

**Table S1**. Repeat sequence content of sequenced Cutaneotrichosporon and reference model yeasts

**Table S2**. Predicted position of exons of secondary barcode genes by tBLASTn. The protein sequences of S. cerevisiae were used as queries. Split alignments (corresponding to introns) that are not common to all strains are shown in bold.

**Table S3**. The positions of ITS sequences used for identity calculations in the genome assembly. If there are multiple identical sequences, up to five are shown. The sequence of Cr. amylolentus was used as deposited in GenBank (not extracted from assembly) because no ITS sequence was found in the assembly.

**Table S4**. Sorted chromosomes of reference Cryptococcus genomes for the calculation of LCBs

**Table S5**. Accession numbers for genome assembly

## Data Availability

The sequencing reads, assembly genomes, and annotations have been deposited in the DDBJ database under BioProject accession PRJDB15446 (https://ddbj.nig.ac.jp/resource/bioproject/PRJDB15446). Sequence reads were submitted with accession numbers DRA016334 to DRA016337 (can be found at https://ddbj.nig.ac.jp/search). Assembly genome sequences are available from both DDBJ and NCBI GenBank under accession numbers AP028204 to AP028247 (https://www.ncbi.nlm.nih.gov/nuccore/?term=AP028204%3AAP028247+%5Baccession%5D) and BTCM01000001 to BTCM01000012 (https://www.ncbi.nlm.nih.gov/nuccore/BTCM00000000.1). Details are listed in the Table S5. The script to draw chromosome synteny was published on GitHub (https://github.com/yk-kobayashi/syntplot).

## Abbreviations

ANI: Average nucleotide identity
DDH: DNA-DNA hybridization
GBDP: Genome blast distance phylogeny
ITS: Internal transcribed spacer
LCB: Locally colinear block
rDNA: Ribosomal DNA
