# Correction: Chromosome-level genome assemblies of *Cutaneotrichosporon* spp. (Trichosporonales, Basidiomycota) reveal imbalanced evolution between nucleotide sequences and chromosome synteny

**DOI:** 10.1186/s12864-023-09745-z

**Published:** 2023-10-30

**Authors:** Yuuki Kobayashi, Ayane Kayamori, Keita Aoki, Yuh Shiwa, Minenosuke Matsutani, Nobuyuki Fujita, Takashi Sugita, Wataru Iwasaki, Naoto Tanaka, Masako Takashima

**Affiliations:** 1 Laboratory of Yeast Systematics, Tokyo NODAI Research Institute (TNRI), Tokyo University of Agriculture, 1-1-1 Sakuragaoka, Setagaya, Tokyo 156-8502 Japan; 2 Department of Molecular Microbiology, Faculty of Life Sciences, Tokyo University of Agriculture, 1-1-1 Sakuragaoka, Setagaya, Tokyo 156-8502 Japan; 3 NODAI Genome Research Center, Tokyo University of Agriculture, 1-1-1 Sakuragaoka, Setagaya, Tokyo 156-8502 Japan; 4 Department of Microbiology, Meiji Pharmaceutical University, 2-522-1 Noshio, Kiyose, Tokyo 204-8588 Japan; 5 Department of Integrated Biosciences, Graduate School of Frontier Sciences, The University of Tokyo, Kashiwa, Chiba 277-0882 Japan

**Correction: BMC Genomics 24, 609 (2023)**

**https://doi.org/10.1186/s12864-023-09718-2**

Following publication of the original article [1], it was reported that part of the figure captions for Figs. 1, 2, 3, 4 and 5 were mistakenly inserted into the article body.

Part of the caption of Fig. 1 appeared in the sub-section “Sequencing and assembly results” and was processed as the paragraph directly preceding the beginning with “The self-synteny plot of the Cutaneotrichosporon genome showed no obvious centromeric repeats …”

Part of the caption of Fig. 2 appeared as the final paragraph of the sub-section “Comparison of nuclear genomes.”

Part of the caption of Fig. 3 appeared as the final paragraph of the sub-section “Quantification of differences in genomes using different criteria.”

The captions of Fig. 4 and 5 appeared as the final two paragraphs of the sub-section “Genes and synteny of mitochondrial genomes.”

The correct Figs. 1-5 with their caption﻿s are given in this Correction article and the original article [1] has been updated.

**Fig. 1:**
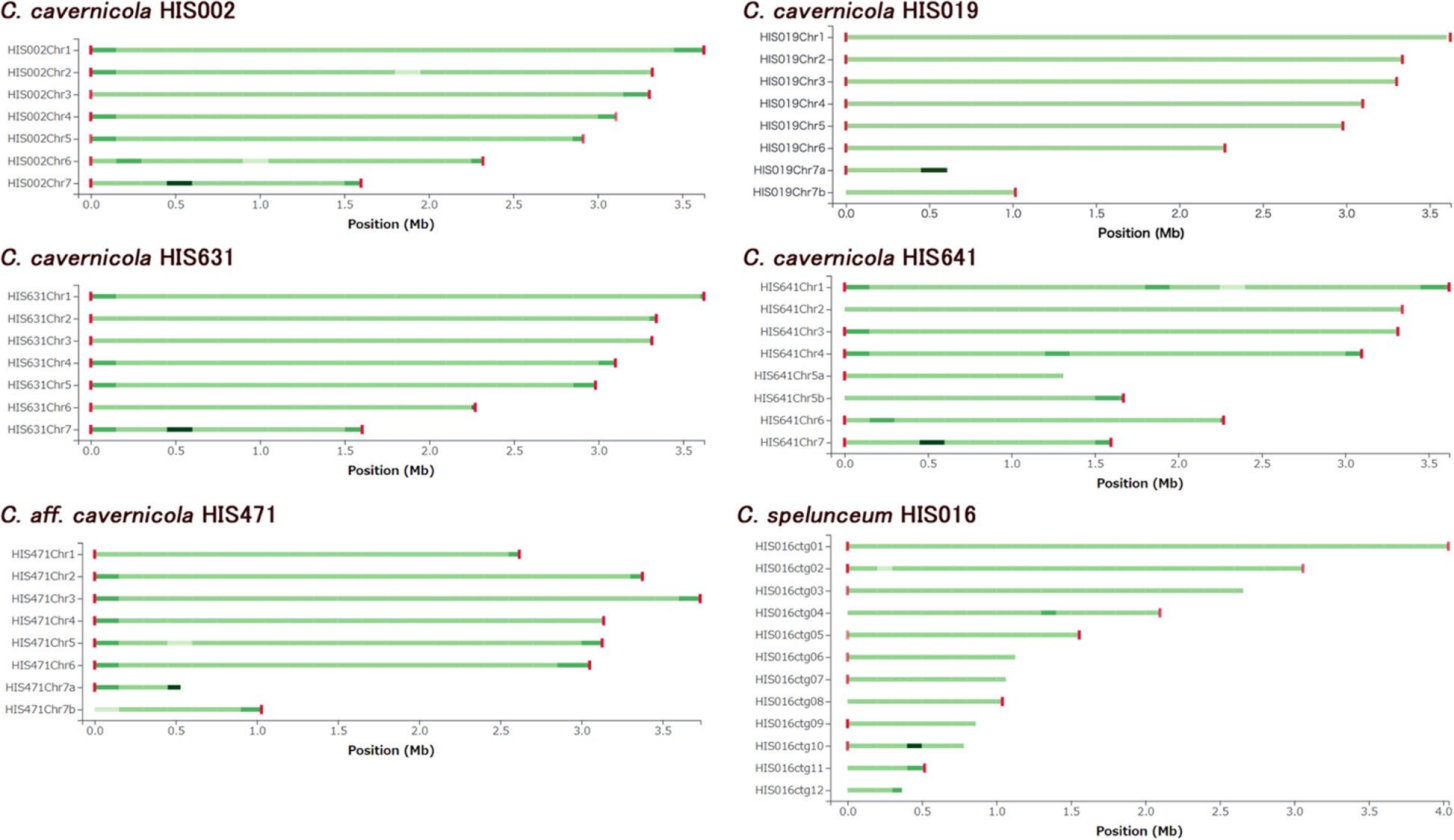
Chromosome continuity of genomes assemblies. Telomere sequence and sequencing depth was illustrated using Tapestry 1.0.0. Red rectangles at the termini stand for telomere repeat sequences (CCCCTAA/ TTAGGGG). The intensity of the green lines indicates the depth of sequencing reads. The dark-coloured region on Chr.7 in the genomes of HIS002, HIS019, HIS631, HIS641, and HIS471, and on ctg.10 in the genome of HIS016 correspond to rDNA repeats

**Fig. 2:**
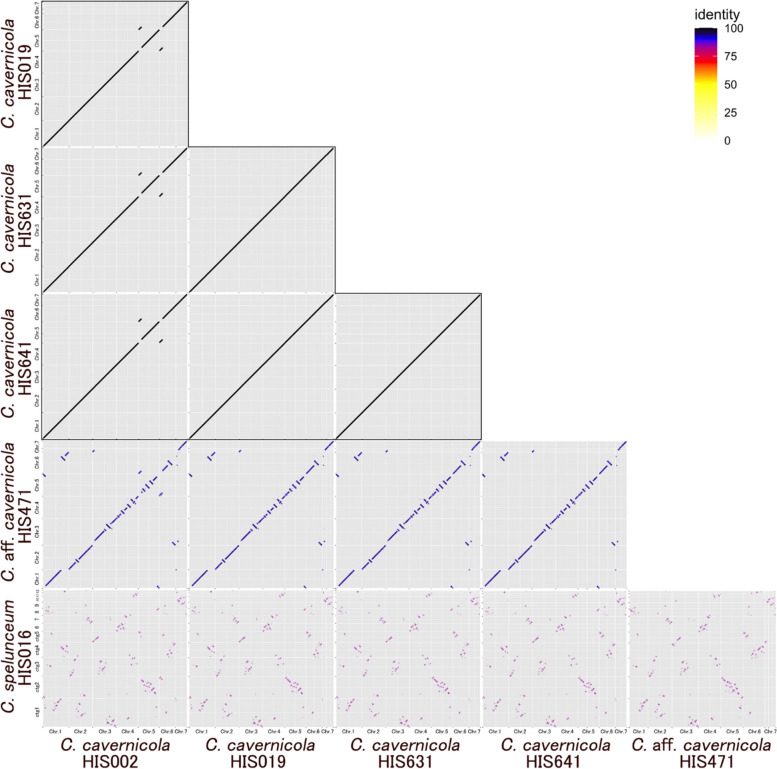
Plots of chromosome synteny based on pairwise BLASTN alignment among *Cutaneotrichosporon* strains. The line colour reflects the percentage of nucleotide identity in the alignment as shown in the legend

**Fig. 3:**
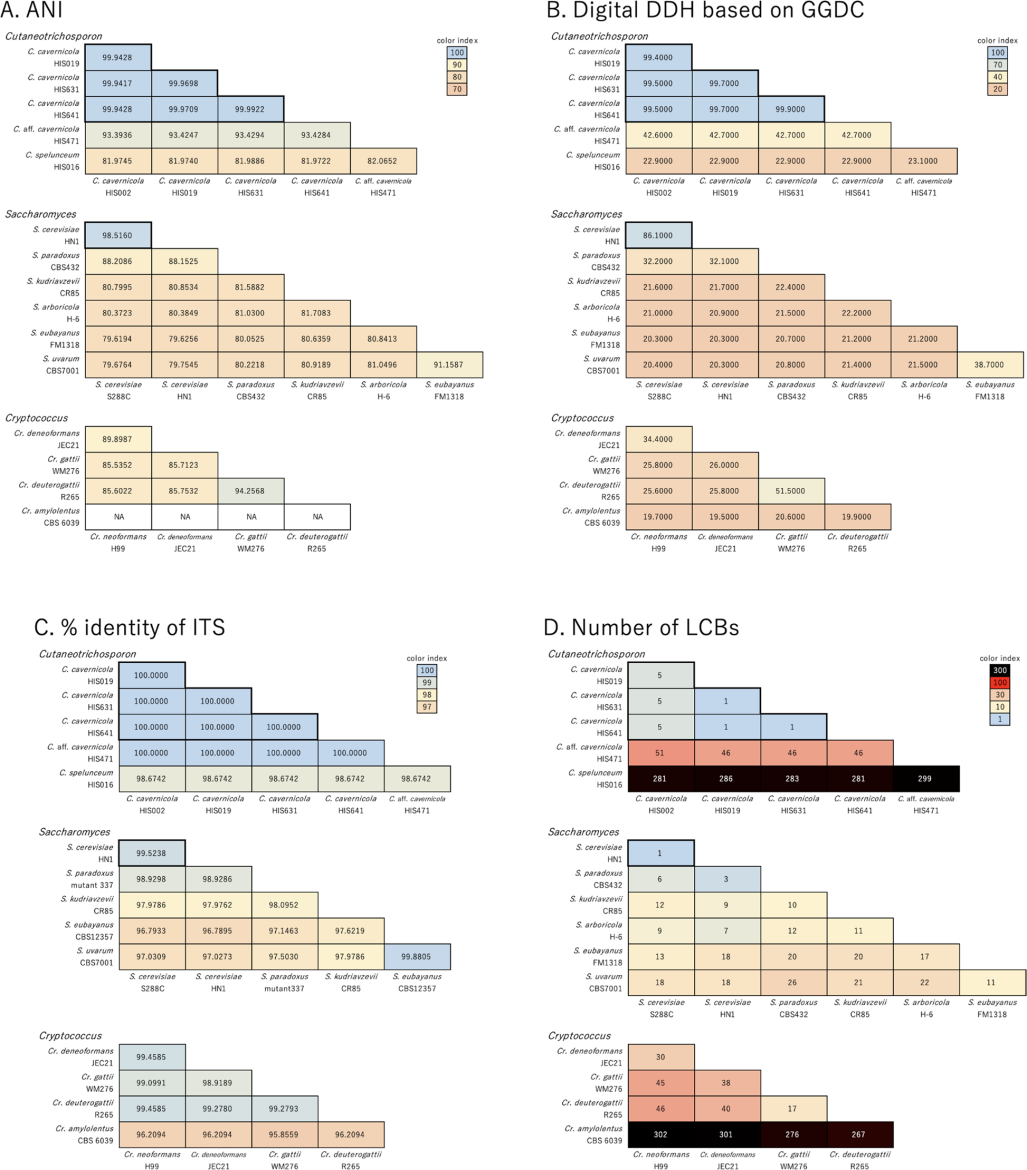
Genome similarities based on multiple criteria among *Cutaneotrichosporon* strains compared with reference *Saccharomyces* and *Cryptococcus*. Thick-bordered areas in the *Cutaneotrichosporon* and *Saccharomyces* panels indicate intraspecific comparisons among the *C. cavernicola* standard strains and *S. cerevisiae*, respectively. Box colours identify identical genomes (blue) and the most distant interspecific comparison (orange) in *Saccharomyces*. **A**; ANI score. **B**; GBDP score calculated with GGDC. The scores for formula 2 are shown according to the recommendation in Henz et al. [9], and scores by all three formulae are shown in Fig. S6. **C**; Percentage identity in the ITS sequence. **D**; Number of LCBs with a minimum weight of 10 kb

**Fig. 4:**
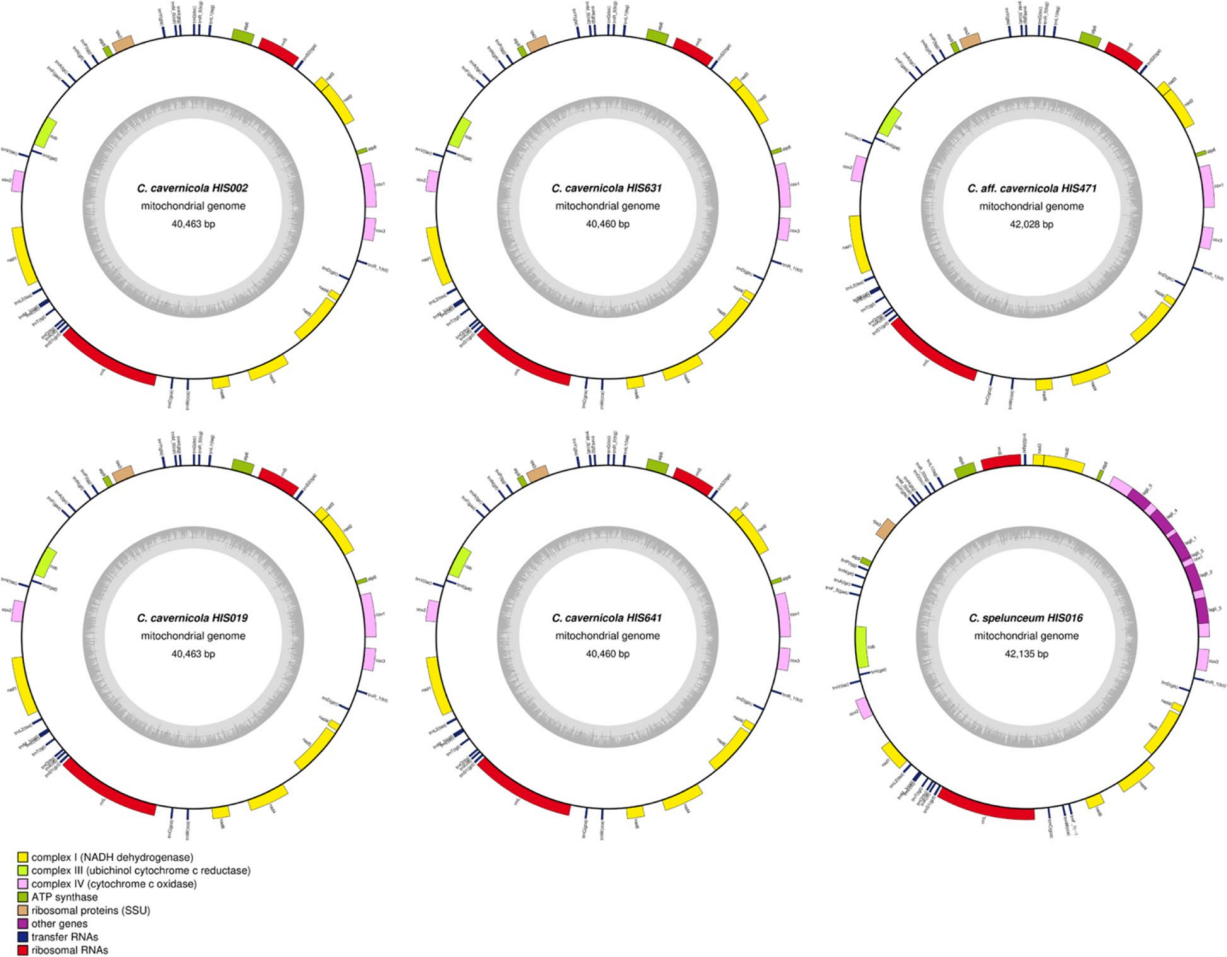
Mitochondrial genomes of *Cutaneotrichosporon* strains. Genes projecting outward from the outer circles indicate genes transcribed in the forward direction; genes projecting inward from the outer circles indicate genes transcribed in the reverse direction. Gene families are identified by colour as shown in the legend. The inner circles represent the GC content of the sequences

**Fig. 5:**
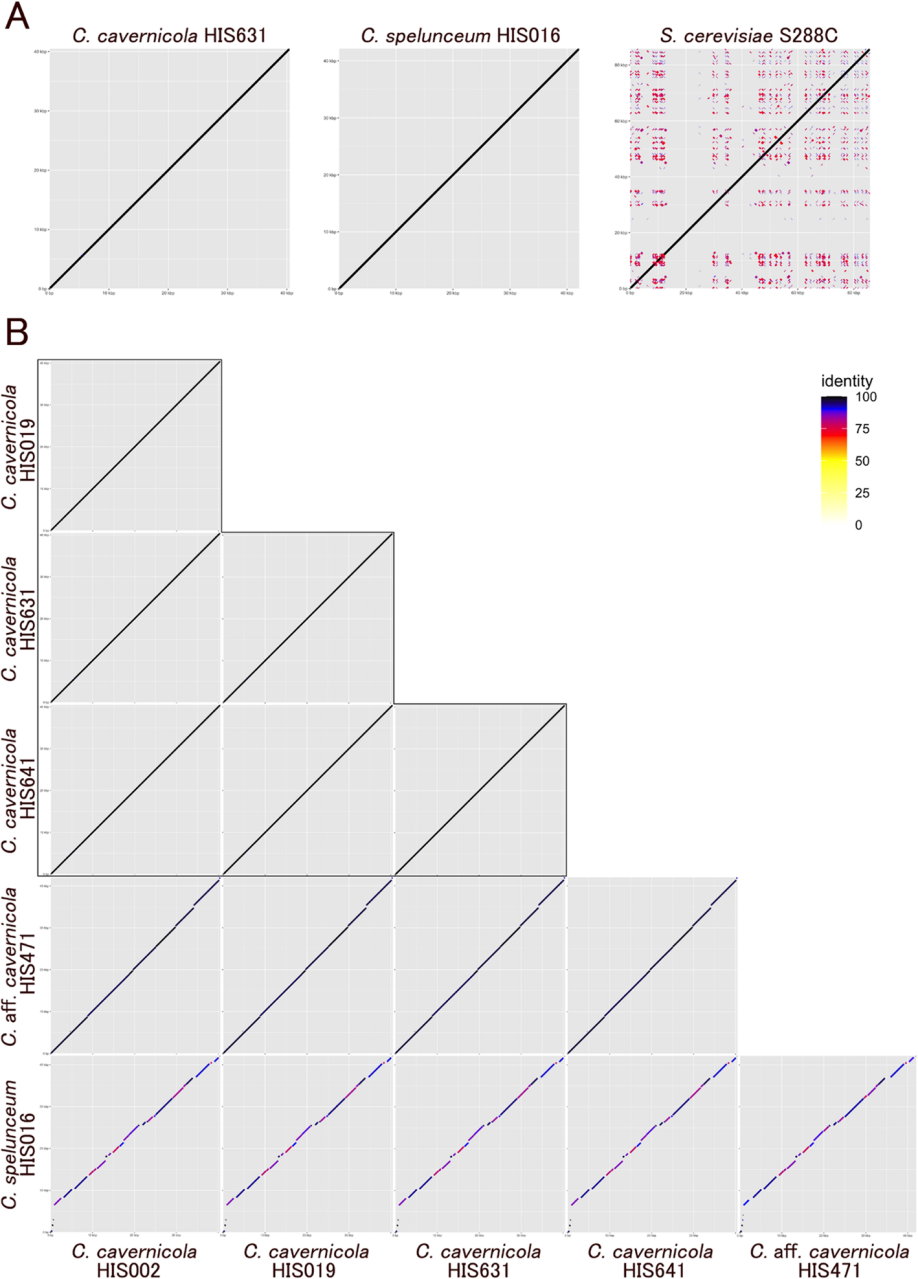
Plots of mitochondrial genome synteny based on pairwise BLASTN alignments. Line colour reflects the percentage of nucleotide identity in the alignment as shown in the legend. **A**; Self synteny of C. cavernicola HIS631, C. spelunceum HIS016, and the reference S. cerevisiae S288C. **B**; Pairwise synteny plots among Cutaneotrichosporon mitogenomes
