# Supplementary material for: Chromosome-level genome assemblies of Cutaneotrichosporon spp. (Trichosporonales, Basidiomycota) reveal imbalanced evolution between nucleotide sequences and chromosome synteny
Source: BMC Genomics. 2023 Oct 11;24:609. doi: 10.1186/s12864-023-09718-2 (PMC10568926; doi:10.1186/s12864-023-09718-2)
Supplement: Supplementary file 1 — Fig. S1. Characteristics of Cutaneotrichosporon sp. HIS471. A, B; Microscopic images of strain HIS471. Cells were incubated on corn meal agar medium (Nissui, Japan) at 20 °C for four days, and observed using a BX53 compound microscope with an UPlansXApo 40? objective lens (Olympus, Japan). Bars; 20 m. C, D; Pairwise alignment of strain HIS019 (C. cavernicola type) and strain HIS471. C and D represent the ITS region and the D1/D2 region, respectively. The polymorphic sites are indicated by arrows. E, F; The possible RNA secondary structure of D1/D2 region of HIS019 (E) and HIS471 (F) predicted with the minimum free energy (MFE) method. Fig. S2. Self-synteny plots of C. cavernicola and Cryptococcus neoformans genomes. Self-synteny plots of C. cavernicola HIS019 and reference Cryptococcus neoformans H99 (GCA_011801205.1) genomes. The plot of the C. cavernicola genome shows no visible repeats, in contrast to the plot of the Cr. neoformans genome, which shows repetitive palindromes (which appear as “X” in the figure) corresponding to the centromeres in each chromosome. Fig. S3. Mauve alignment of Cutaneotrichosporon genomes. Chromosome synteny of Cutaneotrichosporon visualized with Mauve 2015-2-25. Each coloured block represents locally colinear blocks (LCBs). Fig. S4. Chromosome synteny of Saccharomyces. BLASTN-based chromosome synteny of the reference model yeast Saccharomyces. Line colour reflects the percentage of nucleotide identity in the alignment as shown in the legend. Fig. S5. Alignment of ITS sequences of Cutaneotrichosporon strains. Multiple alignment of ITS sequences of Cutaneotrichosporon strains. The ITS sequences were extracted from assembly genomes with the SeqKit amplicon. The polymorphic sites are indicated by arrows. Fig. S6. GBDP scores calculated by all three formulae of GGDC. The GBDP scores among Cutaneotrichosporon and among reference Saccharomyces and Cryptococcus calculated by using three formulae with the genome-to-genome distance calculator ( [file 12864_2023_9718_MOESM1_ESM.docx]

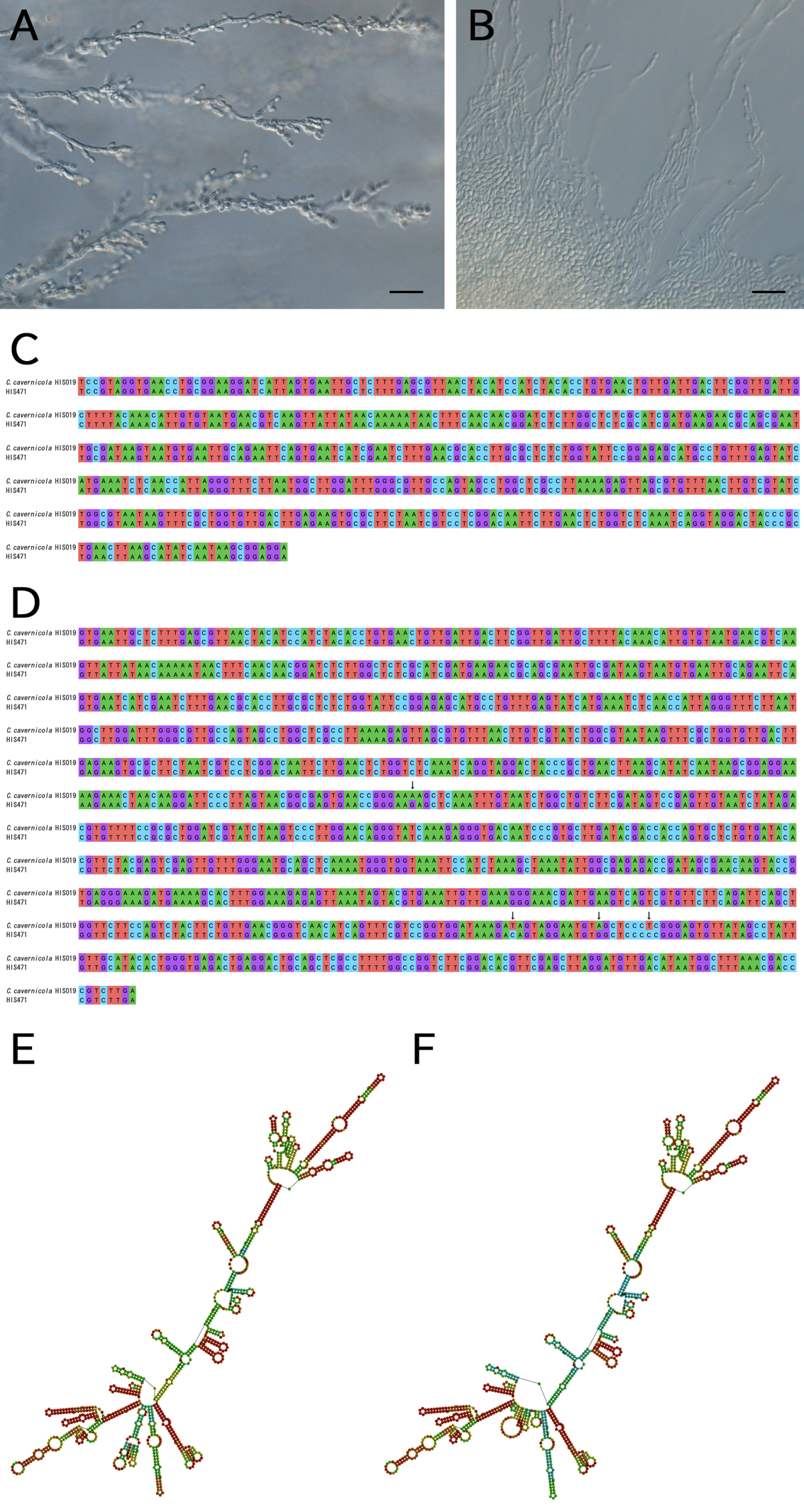


**Fig. S1. Characteristics of *Cutaneotrichosporon* sp. HIS471**

A, B; Microscopic images of strain HIS471. Cells were incubated on corn meal agar medium (Nissui, Japan) at 20 °C for four days, and observed using a BX53 compound microscope with an UPlansXApo 40× objective lens (Olympus, Japan). Bars; 20 μm. C, D; Pairwise alignment of strain HIS019 (*C. cavernicola* type) and strain HIS471. C and D represent the ITS region and the D1/D2 region, respectively. The polymorphic sites are indicated by arrows. E, F; The possible RNA secondary structure of D1/D2 region of HIS019 (E) and HIS471 (F) predicted with the minimum free energy (MFE) method.


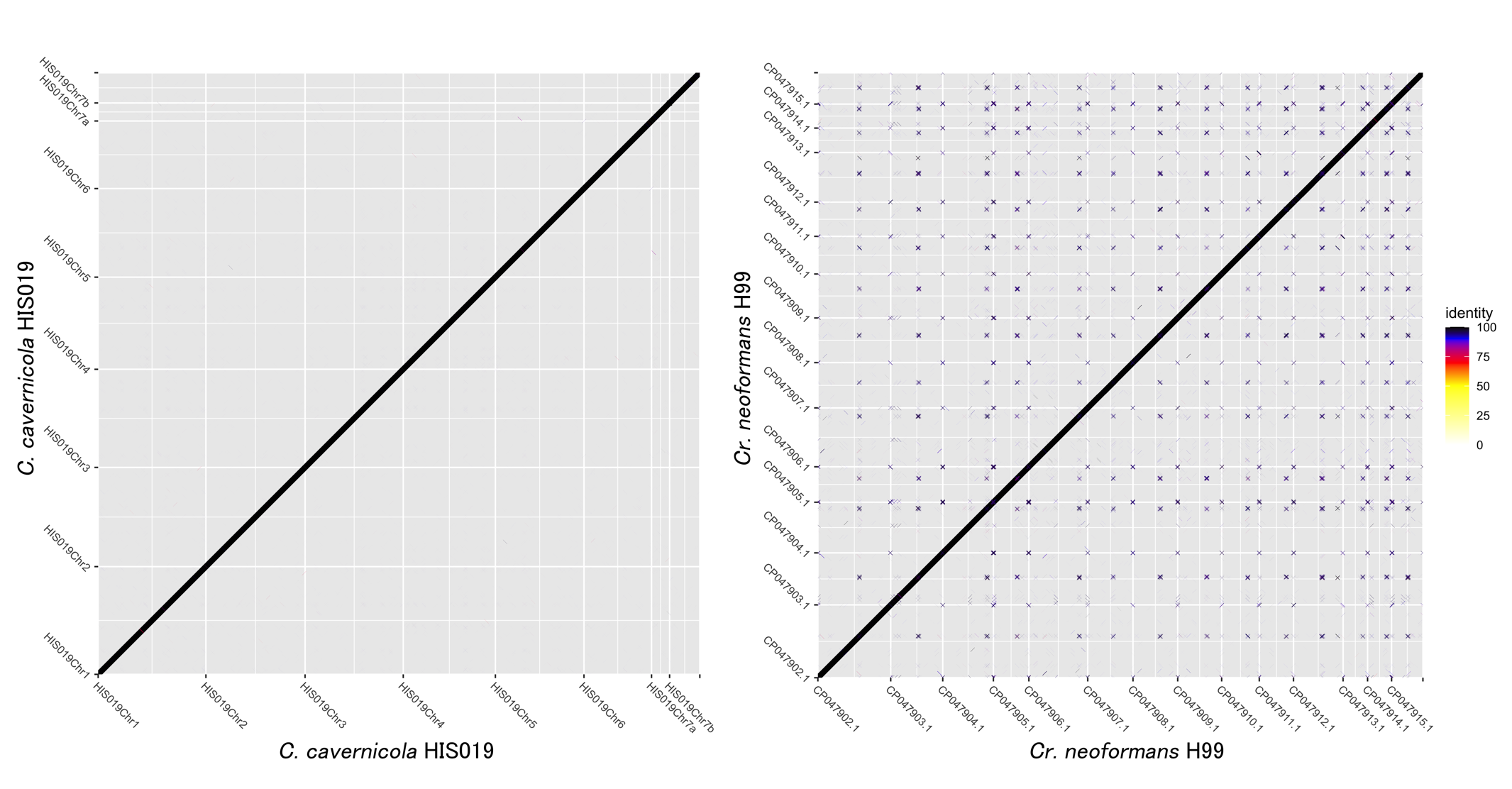


**Fig. S2. Self-synteny plots of *C. cavernicola* and *Cryptococcus neoformans* genomes**

Self-synteny plots of *C. cavernicola* HIS019 and reference *Cryptococcus neoformans* H99 (GCA_011801205.1) genomes. The plot of the *C. cavernicola* genome shows no visible repeats, in contrast to the plot of the *Cr. neoformans* genome, which shows repetitive palindromes (which appear as “X” in the figure) corresponding to the centromeres in each chromosome.


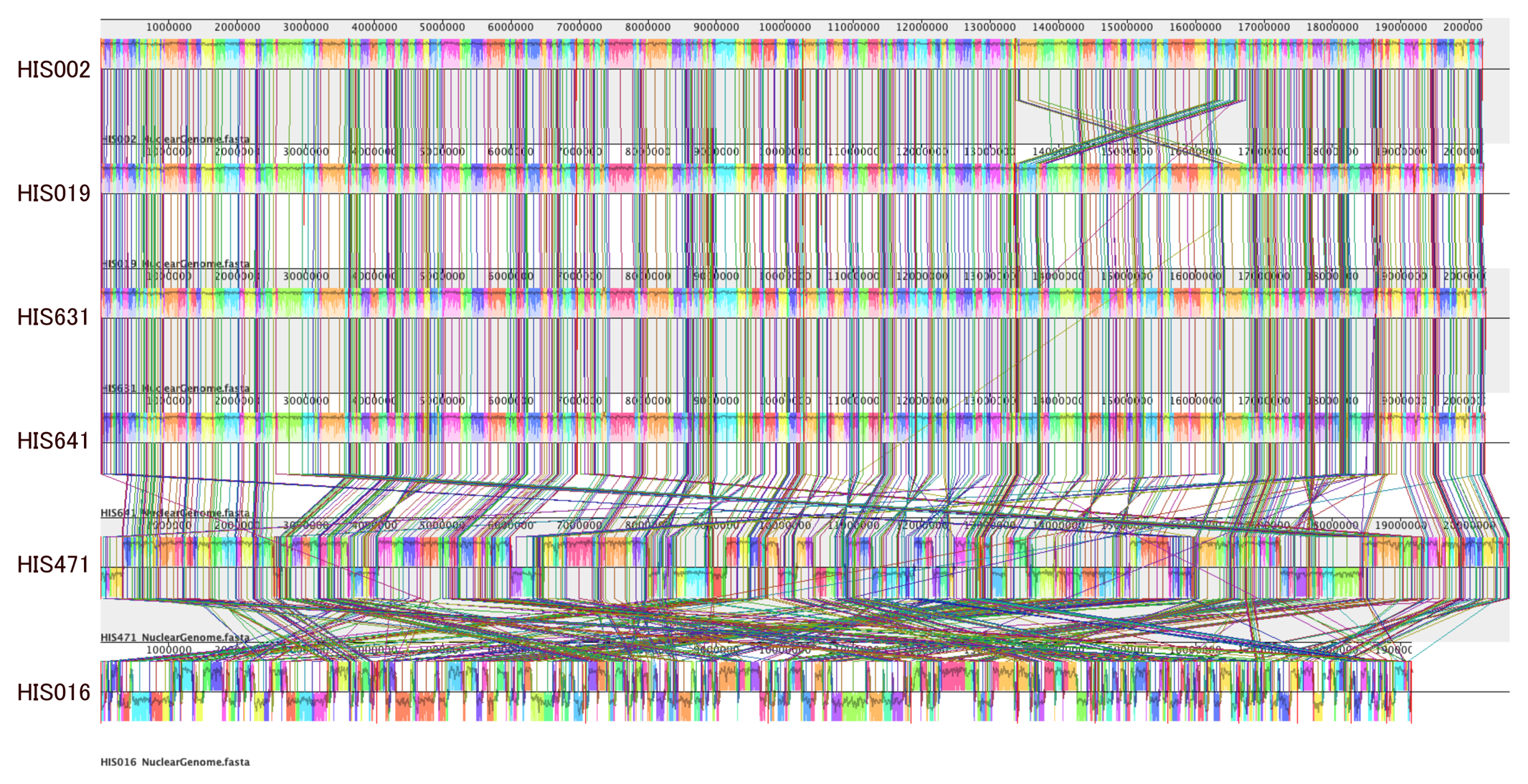


**Fig. S3. Mauve alignment of *Cutaneotrichosporon* genomes**

Chromosome synteny of *Cutaneotrichosporon* visualized with Mauve 2015-2-25. Each coloured block represents locally colinear blocks (LCBs).


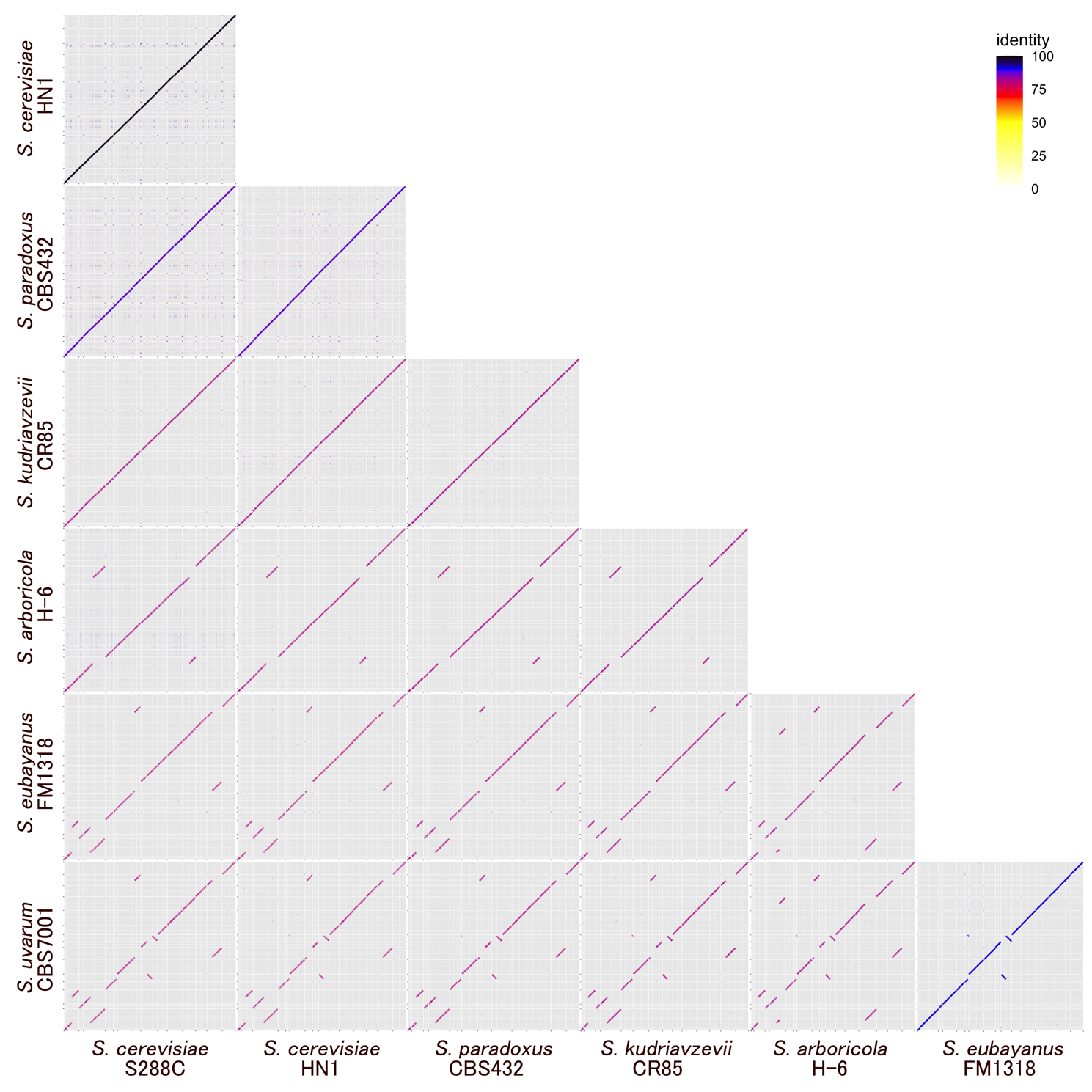


**Fig. S4. Chromosome synteny of *Saccharomyces***

BLASTN-based chromosome synteny of the reference model yeast *Saccharomyces*. Line colour reflects the percentage of nucleotide identity in the alignment as shown in the legend.


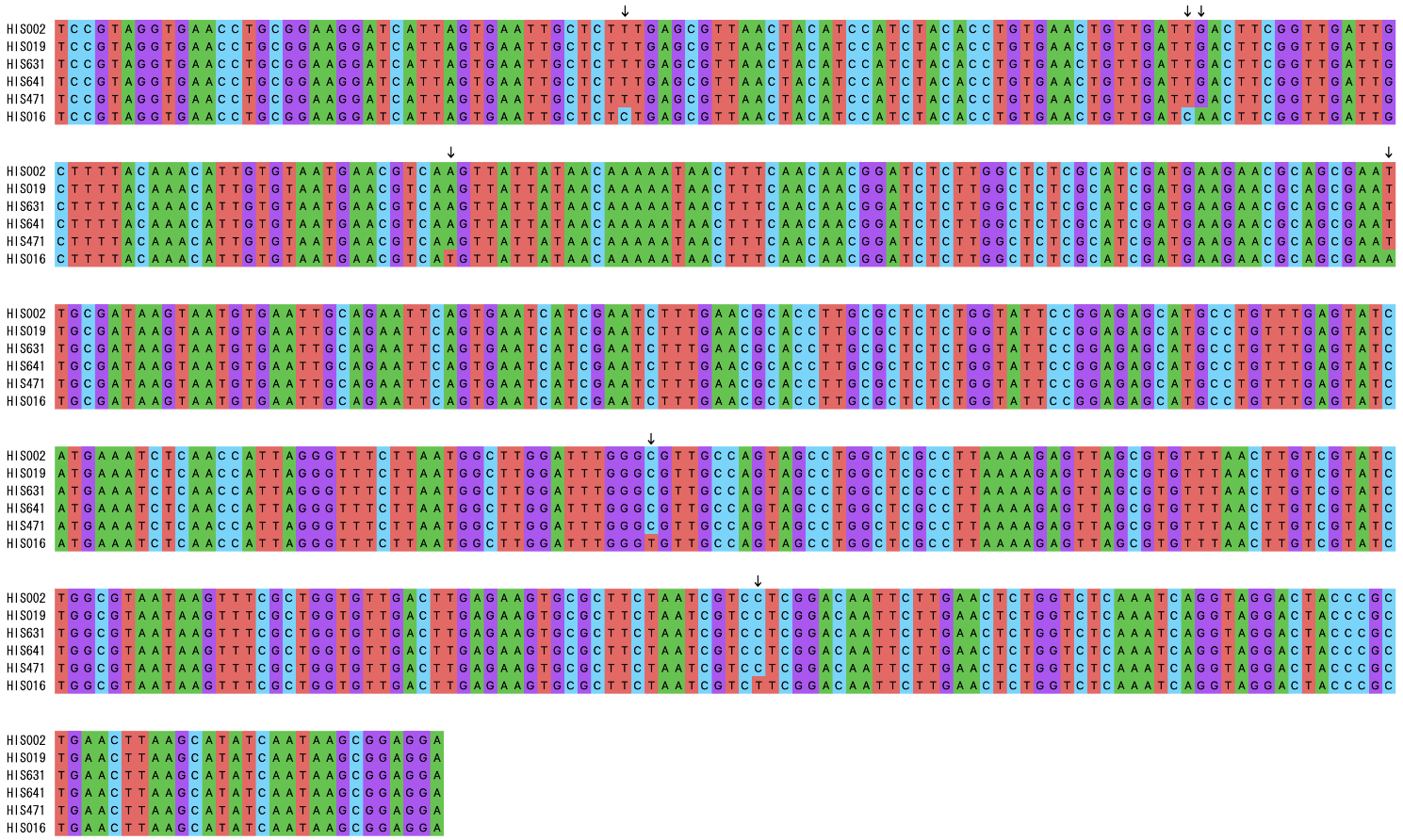


**Fig. S5. Alignment of ITS sequences of *Cutaneotrichosporon* strains**

Multiple alignment of ITS sequences of *Cutaneotrichosporon* strains. The ITS sequences were extracted from assembly genomes with the SeqKit amplicon. The polymorphic sites are indicated by arrows.


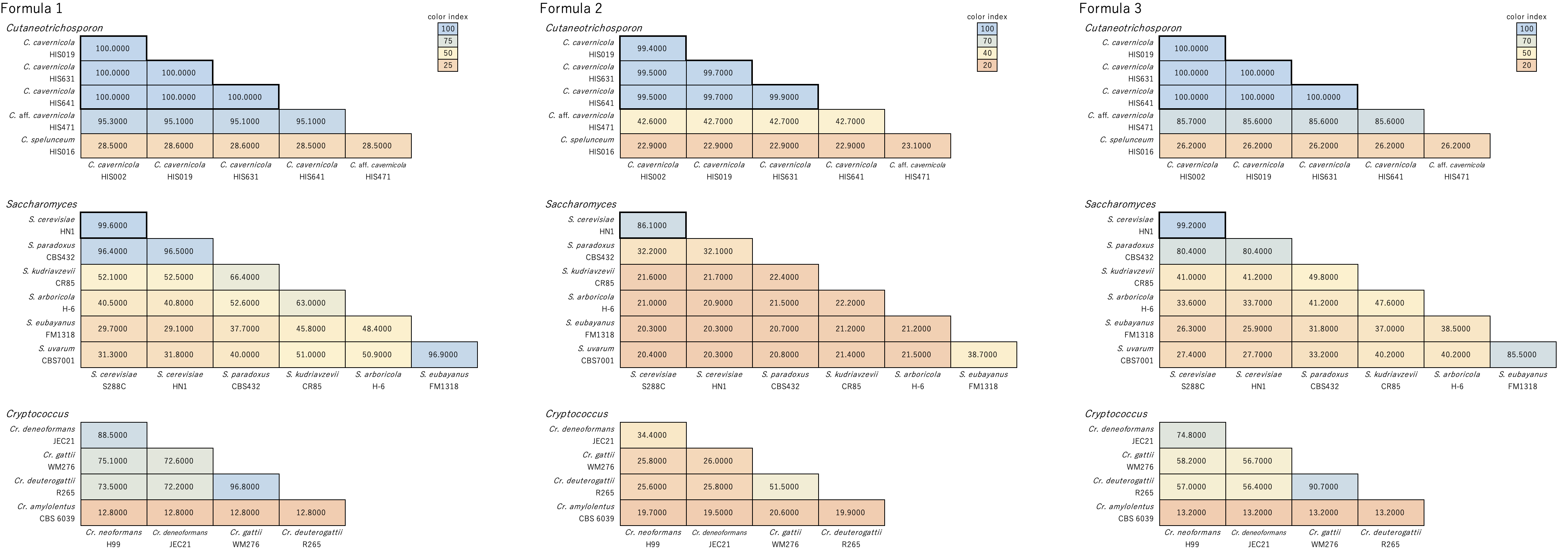


**Fig. S6. GBDP scores calculated by all three formulae of GGDC**

The GBDP scores among *Cutaneotrichosporon* and among reference *Saccharomyces* and *Cryptococcus* calculated by using three formulae with the genome-to-genome distance calculator (GGDC). Blue boxes represent identical genomes and orange boxes represent the most distant interspecific comparison in the reference genomes.


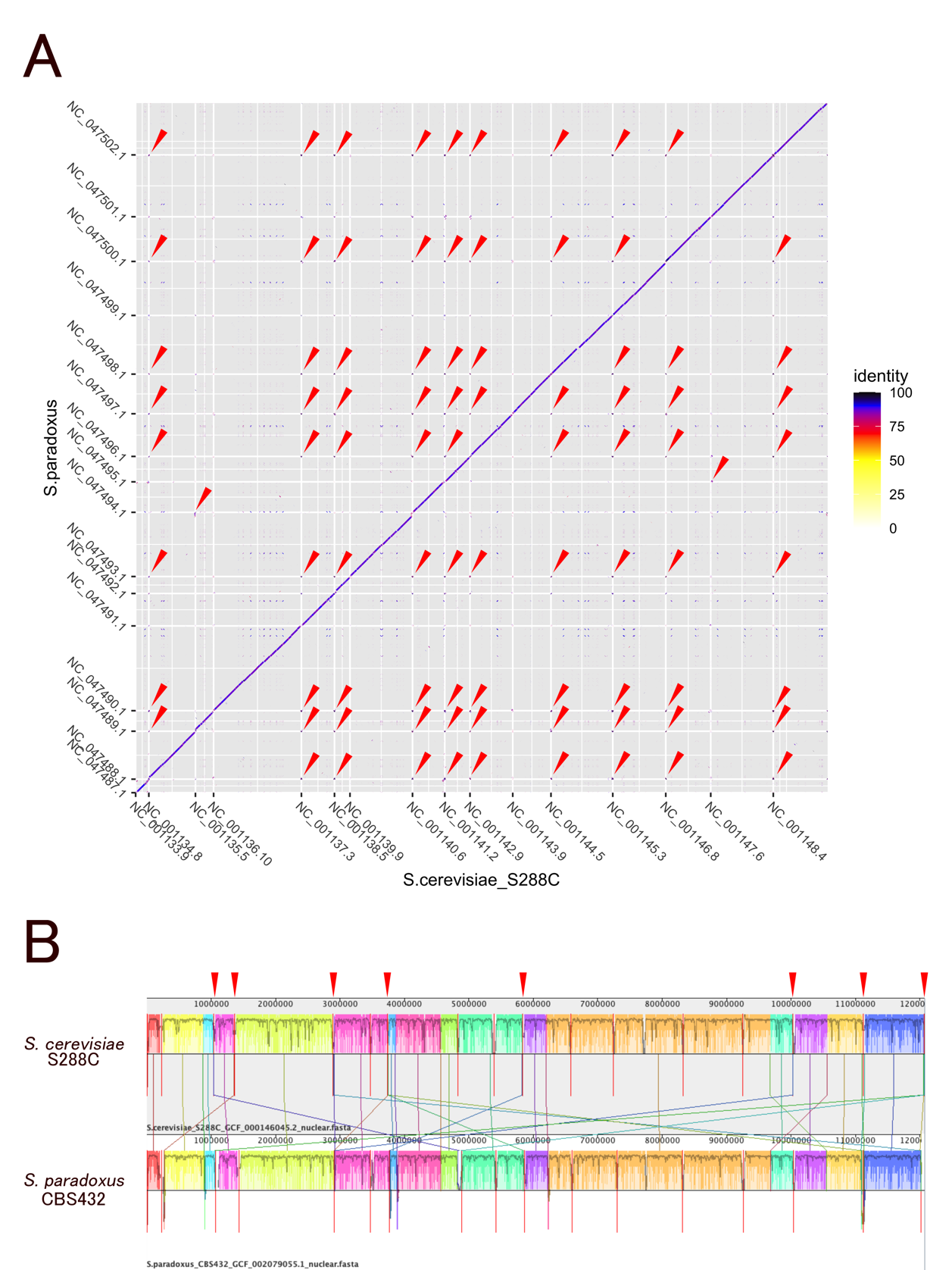


**Fig. S7. Satellite syntenies in reference *Saccharomyces* caused by repetitive sequences**

Chromosome synteny between reference *S. cerevisiae* and *S. paradoxus*. A; BLAST-based synteny visualization. Red arrowheads represent satellite syntenies caused by telomeric repeats. B; Synteny visualized with Mauve 2015-2-25 alignment. Red arrowheads represent LCBs from satellite syntenies of repetitive sequences.
